# The efficacy of dapagliflozin combined with hypoglycemic drugs in treating type 2 diabetes: protocol for meta-analysis of randomized controlled trials

**DOI:** 10.1186/2046-4053-2-103

**Published:** 2013-11-13

**Authors:** Yu-nan Sun, Yi Zhou, Xi Chen, Wen-si Che, Siu-wai Leung

**Affiliations:** 1 State Key Laboratory of Quality Research in Chinese Medicine, Institute of Chinese Medical Sciences, University of Macau, Macao, China; 2 School of Informatics, University of Edinburgh, Edinburgh, United Kingdom

**Keywords:** Systematic review, Dapagliflozin, Type 2 diabetes, Meta-analysis

## Abstract

**Background:**

Dapagliflozin is a first-in-class oral sodium glucose co-transporter 2 (SGLT2) inhibitor. It is often used in combination with conventional anti-diabetic drugs such as metformin, glimepiride, and insulin in treating type 2 diabetes (T2D). It not only reduces glucose reabsorption in the kidney but also increases renal glucose excretion. Some studies found the actions of dapagliflozin independent of insulin and free from risk of weight gain. This meta-analysis aims to evaluate whether dapagliflozin is synergistic with other anti-diabetic drugs without risk of weight gain.

**Methods/Design:**

This meta-analysis will include the randomized controlled trials (RCT) evaluating the efficacy of dapagliflozin as an add-on drug in treating T2D for >8 weeks with the outcome measures glycosylated hemoglobin (HbA1c), fasting plasma glucose (FPG) and body weight. Information of relevant RCTs will be retrieved from major databases including PubMed, Cochrane Library, Embase, ClinicalTrials.gov, and Google Scholar according to a pre-specified search strategy. Google and manual search will find other unpublished reports and supplementary data. Eligible RCTs will be selected according to pre-specified inclusion and exclusion criteria. Data will be extracted and input into a pre-formatted spreadsheet. The Cochrane risk of bias tool will be used to assess the quality of the eligible RCTs. Meta-analysis based on the random-effects model will be conducted to compare the changes of HbA1c (%), FPG (mmol/L), and body weight (kg) between dapagliflozin arm and placebo arm. Publication bias will be evaluated with a funnel plot and the Egger’s test. Heterogeneity will be assessed with the I^2^ statistics. Sensitivity analysis will be conducted on follow-up periods. The evidential quality of the findings will be assessed with the GRADE profiler.

**Discussion:**

The findings of this meta-analysis will be important to clinicians, patients, and health policy-makers regarding the use of dapagliflozin in T2D treatment.

**Study registration:**

PROSPERO registration number: CRD42013005034

## Background

The efficacy of common anti-diabetic drugs (including metformin, sulfonylureas, nonsulfonylurea secretagogues, alpha glycosidase inhibitors, thiazolidinediones, glucagon-like peptide-1 analog, and dipeptidyl peptidase-4 inhibitors) is insulin-dependent [1]. Their efficacy diminishes with the declines of the function of pancreatic islet β-cells during the progression of type 2 diabetes (T2D) [2]. Sulphonylureas and thiazolidinediones cause weight gain, which will further worsen insulin resistance [3]. It came as no surprise that approximately two-thirds of diabetics in Europe [4] and the United States [5] under conventional treatment could not meet the goal of glucose control. By contrast, as a highly selective inhibitor of sodium glucose co-transporter 2 (SGLT2), dapagliflozin is distinctive in its insulin-independent action on reducing reabsorption of glucose particularly by the proximal tubule in the kidney to eliminate more glucose from plasma into urine [6-8]. Dapagliflozin would enhance glucose control without adverse effects on body weight, blood pressures, and lipids like conventional anti-diabetic drugs [9,10]. These claimed advantages of dapagliflozin would be beneficial for combining conventional anti-diabetic drugs with dapagliflozin in treating T2D. However, these claims were made by individual clinical studies, not well-established by the available systematic reviews and meta-analysis. Three existing meta-analysis reports did not focus on dapagliflozin but addressed the efficacy issues of SGLT2 inhibitors in general [3,11,12]. The only meta-analysis [13] on dapagliflozin in particular still lacked analysis of publication bias and sensitivity to various possible factors as the PRISMA guideline for meta-analysis reporting. Although a subgroup analysis on dapagliflozin monotherapy was available in the meta-analysis [13], it did not provide a specific analysis of the efficacy of dapagliflozin combined with other anti-diabetic drugs. All these four meta-analysis studies were not registered before their conduct. The present meta-analysis aims to evaluate the efficacy of dapagliflozin in combination with conventional anti-diabetic drugs for glucose control as measured by the changes of glycosylated hemoglobin (HbA1c) and fasting plasma glucose (FPG). The body weight data will be analyzed to test whether the claim that dapagliflozin does not affect body weight (that is, no weight gain) was sound across relevant studies.

## Methods/Design

This protocol specifies the conduct and reporting of a systematic review and meta-analysis in compliance with the guideline Preferred Reporting Items for Systematic Reviews and Meta-analyses (PRISMA). The protocol has been registered in the PROSPERO database and assigned an identifier CRD42013005034.

### Date sources

Bibliographical databases for literature search include PubMed, Cochrane Library, Embase, Google Scholar, and ClinicalTrials (http://www.clinicaltrials.gov). Our search strategy will include main keywords ‘dapagliflozin’ and ‘diabetes’ (Appendix). Google search will be conducted to find other RCT information that is not available from bibliographical databases. Manual search will be conducted to track relevant RCTs that are not obviously indexed by normal keywords. Study selection will be documented and summarized in a PRISMA-compliant flowchart (Additional file 1: Figure S1).

### Eligibility criteria

The retrieved studies will be selected according to the checklist in Additional file 2 and the eligibility criteria listed below:

#### Study design

Only RCTs will be included. Observational, cohort, case–control, case series, and laboratory studies will be excluded.

#### Follow up periods

As long enough follow-up time is required for observing changes in HbA1c levels, this meta-analysis will include only the RCTs with follow-up periods >8 weeks.

#### Participants

This meta-analysis will include only the RCTs on adult T2D patients (aged ≥18 years).

#### Interventions

This meta-analysis will include only the RCTs on the efficacy of dapagliflozin combined with conventional anti-diabetic drugs. The RCTs on dapagliflozin monotherapy will be excluded.

#### Comptors

This meta-analysis will include the RCTs employing placebo combined with conventional anti-diabetic drugs as the controls. The RCTs employing only placebo as the control group will be excluded.

#### Outcomes

This meta-analysis will include the RCTs measuring HbA1c, FPG, and body weight as the outcomes. The RCTs without all these three outcomes will be excluded.

### Study selection

At least two reviewers will use the same eligibility evaluation form to evaluate the studies according to the eligibility criteria. Disagreement of their evaluation will be resolved by discussion.

### Data extraction

Data from each included RCT will be extracted by one reviewer and verified by another. In addition to the outcome measures, the following characteristics of the verified RCTs will be extracted: (1) authors (and publication year), (2) interventions (doses of dapagliflozin and the drug used in combination), (3) characteristics of participants, (4) follow-up periods, and (5) conclusion. The extracted data will be tabulated (Additional file 3: Table S1) for further analysis.

### Quality assessment

We will assess the design, execution, and reporting of the included RCTs according to the Cochrane risk of bias tool (Additional file 4: Table S2) [14]. The quality of each RCT will be assessed by one reviewer and verified by another. The quality of evidence will be determined with the Grading of Recommendations Assessment, Development and Evaluation (GRADE) system [15]. The analysis will be conducted with GRADE profiler 3.2.

### Data synthesis and analysis

All statistical analysis will be performed with R 3.0.1 software (http://www.r-project.org/). Meta-analysis based on the random-effects model will be conducted with ‘metaphor’ package [16]. Continuous data such as the changes of HbA1c (%), FPG (mmol/L), and body weight (kg) will be presented as adjusted mean differences with 95% confidence intervals. A subgroup analysis will be conducted according to different drug combinations. The effects of follow-up periods and drug dosages will be assessed by meta-regression.

Publication bias will be evaluated with a funnel plot (that is, a plot of the effect sizes against their standard errors) and Egger’s regression test. Heterogeneity will be assessed with the I^2^ statistic, which is the proportion of total variance observed between the RCTs attributable to differences between RCTs rather than to sampling errors.

### Sensitivity analysis

Sensitivity analysis will be performed to evaluate the robustness of the meta-analysis results. We will exclude the RCTs with some extreme features, for example, long follow-up periods (>24 weeks) or high risks of bias (if any), for sensitivity analyses. We would claim the meta-analysis to be robust or reliable if the sensitivity analysis does not significantly change the final results.

## Discussion

This meta-analysis will synthesize evidence from available RCTs on the efficacy of dapagliflozin in combined use with any conventional anti-diabetic drug, particularly the efficacy measured by the outcomes in HbA1c, FPG, and the body weight. The evidence would be useful to clinicians, patients, and health policy-makers regarding the use of dapagliflozin in T2D treatment.

## Appendix: Search strategy

Database: PubMed.

1. dapagliflozin

2. diabetes

1 and 2 in all fields.

Database: Embase.

1. dapagliflozin

2. diabetes

1 and 2 in abstract, title, keywords.

Database: Cochrane Library.

1. dapagliflozin

2. diabetes

1 and 2 in all Text.

Database: ClinicalTrials.gov.

1. dapagliflozin

## Abbreviations

FPG: Fasting plasma glucose; GRADE: Grading of Recommendations Assessment, Development and Evaluation; HbA1c: Glycosylated hemoglobin; PRISMA: Preferred Reporting Items for Systematic reviews and Meta-analyses; RCT: Randomized controlled trial; SGLT2: Sodium glucose co-transporter 2; T2D: Type 2 diabetes.

## Competing interests

The authors declare that they have no competing interests.

## Authors’ contributions

YNS conceived the study, developed the criteria and searched the literature, and wrote the protocol. YZ assisted in protocol design, managed the literature, selected the studies, performed data analysis, and wrote the protocol. XC and WSC wrote the introduction of this protocol. SWL advised on protocol design and revised the manuscript. All authors read and approved the final manuscript.

## Authors’ information

Yu-nan Sun and Yi Zhou are joint first authors.

## Supplementary Material

Additional file 1: Figure S1 Flow diagram. Click here for file

Additional file 2 Eligibility criteria for screening studies. Click here for file

Additional file 3: Table S1 Basic characteristics of randomized controlled trials included in the systematic review. Click here for file

Additional file 4: Table S2 The Cochrane Collaboration’s tool for assessing risk of bias. Click here for file
