# Supplementary material for: The efficacy of dapagliflozin combined with hypoglycemic drugs in treating type 2 diabetes: protocol for meta-analysis of randomized controlled trials
Source: Syst Rev. 2013 Nov 13;2:103. doi: 10.1186/2046-4053-2-103 (PMC3833641; doi:10.1186/2046-4053-2-103)
Supplement: Additional file 1: Figure S1 — Flow diagram. [file 2046-4053-2-103-S1.doc]

**Additional file 1**

**Figure S1.** Flow diagram.

Records identified through PubMed,

Cochrane Library, Embase and ClinicalTrials.gov

(n = )

Additional records identified through Google and Google Scholar

(n = )

Records after duplicates removed

(n = )

Records screened

(n = )

Records excluded

(n = )

Full-text articles assessed for eligibility

(n = )

Full-text articles excluded, with reasons

(n = )

Studies included in qualitative synthesis

(n = )

Studies included in quantitative synthesis (meta-analysis)

(n = )
