# Supplementary material for: The efficacy of dapagliflozin combined with hypoglycemic drugs in treating type 2 diabetes: protocol for meta-analysis of randomized controlled trials
Source: Syst Rev. 2013 Nov 13;2:103. doi: 10.1186/2046-4053-2-103 (PMC3833641; doi:10.1186/2046-4053-2-103)
Supplement: Additional file 2 — Eligibility criteria for screening studies. [file 2046-4053-2-103-S2.doc]

Additional file 2

Eligibility criteria for screening studies

1. Is this study a randomized controlled trial?

YES _____

NO _____

UNCLEAR _____

2. Is the follow-up period of this study longer than 8 weeks?

YES _____

NO _____

UNCLEAR _____

3. Is the age of T2D patient in the RCT greater than18?

YES _____

NO _____

UNCLEAR _____

4. Is the intervention of this study dapagliflozin combined with conventional anti-diabetic drugs?

YES _____

NO _____

UNCLEAR _____

5. Is the control of this study placebo with conventional anti-diabetic drugs?

YES _____

NO _____

UNCLEAR _____

6. Does this study include any of these three outcomes: HbA1c, FPG, and body weight?

YES _____

NO _____

UNCLEAR _____

If you answer NO to any of these questions, the study will be excluded
