# Supplementary material for: The efficacy of dapagliflozin combined with hypoglycemic drugs in treating type 2 diabetes: protocol for meta-analysis of randomized controlled trials
Source: Syst Rev. 2013 Nov 13;2:103. doi: 10.1186/2046-4053-2-103 (PMC3833641; doi:10.1186/2046-4053-2-103)
Supplement: Additional file 3: Table S1 — Basic characteristics of randomized controlled trials included in the systematic review. [file 2046-4053-2-103-S3.doc]

**Additional file 3: Table S1.** Basic characteristics of randomized controlled trials included in the systematic review.

| Author (year) | Intervention | | Participants baseline characteristic | | | | | Follow-up period (max) | Conclusion |
| --- | --- | --- | --- | --- | --- | --- | --- | --- | --- |
|  | Control | experiment | *n* | Age | HbA1c (%) | BMI or weight (kg/m^2^ or kg) | FPG (mmol/L or mg/dl) |  |  |
| Study 1 |  |  |  |  |  |  |  |  |  |
| Study 2 |  |  |  |  |  |  |  |  |  |
| Study 3 |  |  |  |  |  |  |  |  |  |
| Study 4 |  |  |  |  |  |  |  |  |  |
| …… |  |  |  |  |  |  |  |  |  |
