# Supplementary material for: The efficacy of dapagliflozin combined with hypoglycemic drugs in treating type 2 diabetes: protocol for meta-analysis of randomized controlled trials
Source: Syst Rev. 2013 Nov 13;2:103. doi: 10.1186/2046-4053-2-103 (PMC3833641; doi:10.1186/2046-4053-2-103)
Supplement: Additional file 4: Table S2 — The Cochrane Collaboration’s tool for assessing risk of bias. [file 2046-4053-2-103-S4.doc]

**Additional file 4: Table S2. The Cochrane Collaboration’s tool for assessing risk of bias**

| **Source of bias** | **Review authors’ judgment** | **RCT 1** | **RCT 2** | **RCT 3** | **…** |
| --- | --- | --- | --- | --- | --- |
| Random sequence generation | Selection bias due to inadequate generation of a randomized sequence | low/unclear/high | low/unclear/high | low/unclear/high |  |
| Allocation concealment | Selection bias due to inadequate concealment of allocations before assignment | low/unclear/high | low/unclear/high | low/unclear/high |  |
| Blinding of participants and personnel* | Performance bias due to knowledge of the allocated interventions by participants and personnel during the study | low/unclear/high | low/unclear/high | low/unclear/high |  |
| Blinding of outcome assessment* | Detection bias due to knowledge of the allocated interventions by outcome assessment | low/unclear/high | low/unclear/high | low/unclear/high |  |
| Incomplete outcome data* | Attrition bias due to amount, nature, or handling of incomplete outcome data | low/unclear/high | low/unclear/high | low/unclear/high |  |
| Selective reporting | Reporting bias due to selective outcome reporting | low/unclear/high | low/unclear/high | low/unclear/high |  |

*Assessments should be made for each main outcome or class of outcomes.
